# Patients with Metastatic or Locally Advanced Bladder Cancer Not Undergoing Systemic Oncological Treatment—Characteristics and Long-Term Outcome in a Single-Center Danish Cohort

**DOI:** 10.3390/cancers17071105

**Published:** 2025-03-25

**Authors:** Kira Thorsteinsson, Simone Buchardt Brandt, Jørgen Bjerggaard Jensen

**Affiliations:** 1 Department of Urology, Aarhus University Hospital, 8200 Aarhus, Denmark; simbra@rm.dk (S.B.B.); bjerggaard@skejby.rm.dk (J.B.J.); 2 Department of Clinical Medicine, Aarhus University, 8200 Aarhus, Denmark

**Keywords:** metastatic bladder cancer, locally advanced bladder cancer, systemic treatment, no treatment, untreated, frailty, poor general condition

## Abstract

Background: A subset of patients with locally advanced or metastatic bladder cancer (la/mBC) do not receive systemic oncological treatment. The reasons for refraining from systemic treatment are poorly investigated, as are the characteristics of patients who turn out to be long-term survivors despite lack of treatment. Methods: This retrospective observational cohort study included patients with registration compatible with la/mBC who did not undergo systemic treatment. They were seen at a single tertiary university hospital covering one of 5 Danish regions in the study period from 1 January 2012 until 31 December 2022. Patients were identified through a coding system, and detailed patient information was obtained in the electronic medical records. Results: The initial study population consisted of 472 patients registered with la/mBC according to codes in the patient registries but with no registration of systemic oncological treatment. Out of the total cohort, 159 patients were registered correctly, while the rest of the population were excluded due to misclassification, as 10.8% did not have la/mBC and 55.5% had received oncological treatment. Among correctly registered patients, the median overall survival was 2.6 months (95% CI 0.26; 4.94) from being diagnosed with la/mBC. The most common reasons for lack of treatment were poor general condition (74.2%), patient preference (19.5%), and poor renal function (11.9%). Conclusions: Our study found that a significant amount of patients in a cohort of suspected la/mBC not undergoing treatment either received oncological treatment or did not have la/mBC, demonstrating a misclassification in the system. This should be taken into consideration when evaluating data about survival time for this patient group. The poorest survival of untreated la/mBC is associated with a high tumor burden and specifically liver metastasis.

## Simple Summary

Bladder cancer is a common type of cancer, and in its metastatic form, it has a poor prognosis. Despite treatment options, a subset of patients with metastatic bladder cancer do not receive systemic oncological treatment. The aim of this study was to explore the reasons for abstaining from treatment. Patients were identified through a coding system, and patient data were retrieved from the electronic health records system. Among a total of 159 patients with metastatic bladder cancer, the most common reasons were poor general condition, patient preference, and decreased renal function. Furthermore, the study sought to investigate the characteristics of the patients who turned out to be long-term survivors despite the lack of treatment. The study can bring valuable information to the treatment decision and is underscoring the need for a nuanced approach and patient-tailored treatment strategies to improve the overall survival from metastatic bladder cancer.

## 1. Introduction

Bladder cancer is among the top ten most prevalent cancer diagnoses globally, with approximately 550,000 new cases annually [1,2]. In Denmark, about 2000 patients are diagnosed with bladder tumors each year, and around half of these have invasive tumors, whereas roughly half of them are muscle-invasive bladder cancer (MIBC) [3]. Up to 20% of patients present with metastatic or unresectable disease [4,5]. Moreover, approximately 50% of the patients who undergo curative, intended local radical treatment for MIBC will subsequently develop metastases [6,7]. Metastases originating from bladder cancer commonly manifest in lymph nodes, bones, lungs, and liver [8].

Patients with locally advanced or metastatic bladder cancer (la/mBC), including relapse after prior cystectomy or inoperable relapse after radiotherapy, are generally considered incurable. Theoretically, these patients are only candidates for palliative systemic oncological treatment [6,9].

Previous studies have found that more than 50% of patients diagnosed with la/mBC receive no systemic treatment [10,11,12,13]. The decision to not initiate treatment is often based on several factors like poor performance status, comorbidities, and patient preferences, but literature about the reasons for foregoing treatment is sparse. Additionally, the prevalence and characteristics of patients not undergoing treatment have previously only sparsely been described [14,15].

Interestingly, a proportion of patients with metastatic disease are found to have a fair overall survival even without relevant systemic treatment [16]. The underlying reasons for this unexpected survival trend remain inadequately investigated.

This retrospective single-center observational cohort study was conducted in order to investigate the prevalence, characteristics, and overall survival for patients with metastatic or locally advanced bladder cancer who refrained from receiving systemic oncological treatment. It raises questions about the found fair overall survival time. Additionally, the study sought to explore the reasons for why treatment was withheld and thereby provide valuable insights into the clinical landscape of untreated la/mBC patients.

## 2. Materials and Methods

### 2.1. Data Sources and Study Population

The patients in this study were diagnosed with bladder cancer and treated at the Department of Urology in Aarhus University Hospital between January 2012 and December 2022. Patients with suspected bladder cancer are referred to the Department of Urology, where the diagnostics are performed. Every patient with a disease suitable for surgery will be treated at the department, while patients who are considered not curable will be referred to the Department of Oncology for assessment. Aarhus University Hospital has approximately 81,000 annual admissions and serves as a regional center for the surgical treatment of bladder cancer, performing around 100 cystectomies each year.

In Denmark, each diagnosis and medical procedure is systematically cataloged within the electronic health records system. These entries are identified by a specific diagnosis code and procedure code, respectively. Furthermore, diagnoses derived from histopathological examinations are categorized by pathologists employing the Systematized Nomenclature of Medicine (SNOMED) and archived in Patobank, which constitutes an integral component of the Danish Pathology Register.

The diagnostic codes for bladder cancer or metastasis combined with a procedure code indicating the absence of treatment with chemotherapy or immunotherapy BWHA*, BOHJI9H*, or BOHJ19J* were used to identify the cohort. The specific diagnostic codes are seen in Table 1.

Danish Data Protection Agency approval was granted before data extraction (1-45-70-104-22). There were no direct patient interactions in relation to the project.

### 2.2. Outcome and Statistical Analysis

The reasoning that led to the decision to not initiate systemic oncological treatment for each patient was documented according to predefined categories. In addition, it was registered if the patient was assessed by an oncologist and if any palliative treatment, besides systemic treatment, was given.

Only patients who had a survival of 3 months or more from diagnosis of locally advanced or metastatic bladder cancer were registered with detailed information about gender, age, height, weight, ASA classification, serum creatinine, and comorbidity status. The date of initial la/mBC diagnosis and specific details regarding metastatic disease were listed. For patients with a known history of urothelial disease, data about their primary disease were recorded as well.

Descriptive analyses included medians and interquartile ranges (IQRs) or 95% confidence intervals (95% CIs) for continuous variables and sample sizes and frequencies for categorical variables.

The Kaplan–Meier method was used for survival analyses. Overall survival (OS) was calculated from the time of la/mBC diagnosis until death from any cause or, if the patient was still alive, the last time the electronic patient record was evaluated.

## 3. Results

A total of 472 patients were identified as the study population; see Table 1.

**Figure 1:**
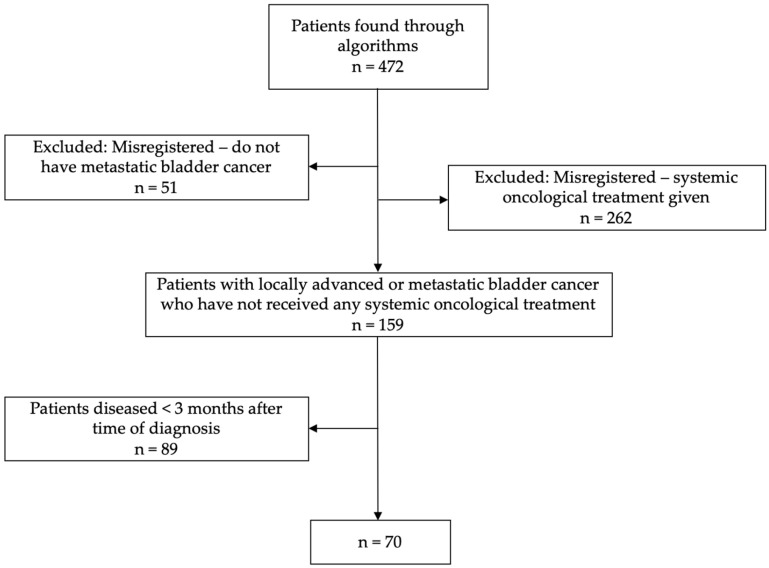
Flowchart showing the population enrolled, reasons for exclusion, and final study population.

Despite their initial classification as not having undergone any form of systemic oncological treatment, 262 (55.5%) of these patients had received chemotherapy and/or immunotherapy subsequent to their date of diagnosis and were excluded from the cohort. Additionally, 51 (10.8%) out of the initial 472 patients were excluded as they did not have locally advanced or metastatic bladder cancer. These patients were predominantly patients with lymph node disease, who had undergone radical cystectomy or radiotherapy without disease relapse during the follow-up time; see the flowchart in Figure 1.

In the entire cohort of 472 patients, a total of 159 patients diagnosed with locally advanced or metastatic bladder cancer had not undergone any form of systemic oncological treatment.

Among the 159 patients, 66.7% were assessed for treatment eligibility by an oncologist, while for the remaining patients, the decision of lack of treatment was made by the urologist or/and the patient itself. Some patients received local palliative treatment, as 29.6% had palliative treatment targeted at metastases and 4.4% targeted the bladder.

The most common reasons for lack of treatment were poor general condition (74.2%), the patients’ personal preferences (19.5%), and poor renal function (11.9%), as outlined in Table 2. For 77% of the patients, one single reason was given, while the remaining 23% of patients had two or three reasons for not undergoing treatment. Among patients surviving less than three months (89 patients), 89.9% were seen in too poor general condition to initiate chemotherapy, and 13.3% had no wish to receive treatment either. The respective numbers for the group surviving for more than three months (70 patients) were 54.3% and 27.1%.

**Figure 2:**
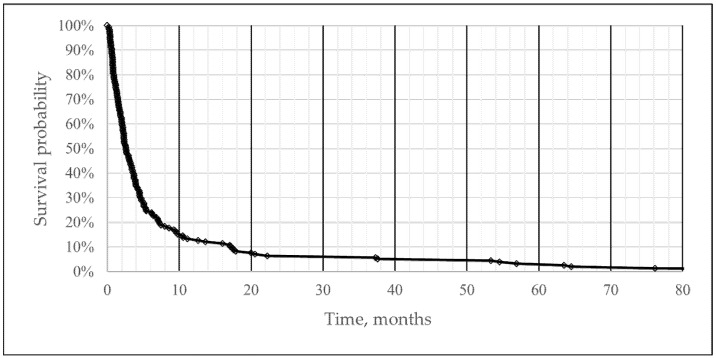
Survival curve illustrating survival from diagnosis of locally advanced bladder cancer or metastatic bladder cancer to death in 159 patients not receiving systemic oncological treatment.

The median OS was 2.6 months (95% CI 0.26; 4.94) after being diagnosed with la/mBC. The 3-, 6-, and 12-month overall survival was 44%, 24%, and 13%. A total of five patients had not died by the end of the study follow-up time. The survival curve is graphically depicted in Figure 2.

From the 159 patients, a subset of 89 individuals died within the initial three months following their diagnosis. Consequently, a group of 70 patients represented patients diagnosed with locally advanced or metastatic bladder cancer who survived more than three months without undergoing any systemic oncological treatment. The characteristics of the patients are presented in Table 3. Among those patients, 54.3% had lymph node metastasis, 28.6% had lung metastases, 25.7% had bone metastases, and 20% had liver metastases.

The majority of the 70 patients, 50 patients (71.4%), were initially diagnosed with local disease and subsequently underwent curatively intended treatment such as cystectomy (72%), radiotherapy (16%), or other forms of local tumor treatment (10%). Furthermore, 15.7% had received neoadjuvant chemotherapy before cystectomy. The metastatic pattern for disease relapse is seen in Table 4 as well as other characteristics of the patients.

## 4. Discussion

This retrospective study was a real-world, community-based investigation of patients diagnosed with la/mBC for an 11-year period. It can be assumed to reflect the general population of la/mBC nationally and in analogous countries globally, rendering the study data relevant and applicable on a larger scale.

Initially, 472 patients were identified in the register; however, 313 patients were misclassified either by diagnosis or by the administered treatment. Consequently, the final study population consisted of 159 patients. Prior to this study, data indicated that a large population of patients with la/mBC without oncological treatment were long-term survivors [16]. This study is questioning that observation, as the prolonged survival time might be caused by the misclassification.

The primary factor contributing to the lack of treatment in the 159 patients was a generally poor physical condition (74.4%). Notably, patients with a compromised general condition did often present with a larger tumor burden compared to those in better physical health. Omission of treatment was seldom exclusively due to comorbidities, except for cases involving decreased renal function, which aligns with existing literature [14,17]. In 19.5% of cases, patients expressed disinterest in systemic treatment, frequently coinciding with assessments of poor general condition rendering them unfit for treatment. The prevailing reasons for lack of treatment observed in this study closely align with findings from comparable research [15]. To our knowledge, no other study reports similar insights into the reasons for abstaining from treatment.

Examining the subgroup of long-term survivors, a notable increase in the proportion of cases where lack of treatment was attributed to the patient’s own wish and age was observed and less to poor general condition, compared to the overall study population.

The long-term survivors had a lower share of liver metastases, aligning with the established understanding that liver metastases serve as an adverse prognostic factor for overall survival [18]. Although the overall metastatic patterns were comparable, long-term survivors generally presented with fewer metastases, while patients with the shortest survival time often displayed severely disseminated disease. Furthermore, local directed treatments for metastases, such as radiotherapy or surgical resection, were more frequent among the patients who ended up with prolonged survival. This indicates the feasibility of local treatment of a single solitary metastasis, a strategy probably ineffective in cases of widespread metastatic disease.

The gender distribution observed in this study closely mirrored the overall la/mBC population in Denmark [19]. Interestingly, the median age at time of diagnosis was notably higher than that of patients initiating chemotherapy (77 vs. 69 years) [19]. The study shed light on the age-dependent increase in the proportion of untreated patients, a predictable observation considering the confluence of declining functional status and age-related comorbidities [12,14,20].

Age was registered only as a contributing factor (9%), suggesting that the decision to forego treatment may be influenced by age-related declines in general physical condition and renal function. While advanced age is conventionally deemed a relative contraindication for chemotherapy-based treatment, the existing evidence does not robustly support the routine exclusion of elderly patients based solely on age [17,21]. Additionally, age-related changes in patient preferences and concerns about treatment toxicity and the risk–benefit ratio of treatment may play a role.

That the median survival time is between 3 and 6 months for metastatic bladder cancer for patients not undergoing systemic oncological treatment but receiving best supportive care has previously been published [9,22,23]. Notably, this study illustrates the poor prognosis associated with la/mBC, highlighting a median survival time of 2.6 months (95% CI 0.26; 4.94) post-diagnosis for patients who do not receive treatment, aligning closely with findings from a comparable Danish study featuring a similar patient population [15]. Independent prognostic factors contributing to poor survival include a poor performance status and visceral metastases [24]. Patients with exclusively lymph node metastases exhibit a more favorable survival outcome [25].

A strength of this study is the real-world design, encapsulating an authentic patient population. The complete inclusion of patients, facilitated by pathology and diagnosis coding practices in Denmark and the utilization of the unique 10-digit personal identification number, emphasizes the robust foundation of this research [26].

The reliance on the electronic patient record system contributes another significant strength, affording detailed information and access to comprehensive patient data from electronic files. However, it is crucial to acknowledge that the electronic patient system, inherent to its retrospective nature, may be susceptible to errors and lack correct classification, potentially introducing a risk of underestimation in comorbidities. An ECOG performance status for every patient would also benefit our understanding of their functional status [21]. Nevertheless, this study is the first to map the long-term survivors after diagnosis of metastatic or locally advanced BC without systemic treatment and additionally the reason for wavering treatment.

## 5. Conclusions

This study found that a considerable number of patients registered with la/mBC and not having received systemic oncological treatment actually underwent treatment or did not have la/mBC, indicating a misclassification in the system. This misclassification might have contributed to the pre-study perception that a larger patient population turned out as long-term survivors.

The study found 159 patients who did forego treatment, which was often due to general poor physical condition, underscoring the need for effective and tolerable treatment. Median survival time was 2.6 months (95% CI 0.26; 4.94) post-diagnosis. Long-term survivors tend to have an overall lower tumor burden and were less likely to have liver metastasis.

This investigation is underscoring the need to advance patient care and tailor treatment strategies more effectively. This will potentially make more patients eligible for treatment and, by that, improve overall survival from la/mBC.

## Figures and Tables

**Table 1:** Distribution of the total of 472 patients found by different codes by the algorithms.

Type of Code	Codes	Definition	*n* (%)
X-diagnosis code	DC679X	Bladder cancer	41 (9)
M-diagnosis code	DC679M	Bladder cancer	96 (20)
Other diagnosis code	DC77, DC78, DC79	Metastasis to the lymphatic system or distant metastasis	239 (51)
SNOMED code	ÆF4530, M8***6, M8***7	Metastasis or recurrence	96 (20)

**Table 2:** Reasons for lack of systemic oncological treatment in 159 patients with locally advanced or metastatic bladder cancer.

Reason	*n* (%)
Poor general condition	118 (74.2)
Patient’s own wish	31 (19.5)
Poor renal function	19 (11.9)
Age	15 (9.4)
Metastasis locally treated (radiotherapy/surgery)	6 (3.8)
Comorbidity	5 (3.1)
Do not tolerate treatment	2 (1.3)
Dialysis	1 (0.6)
Metastasis diagnosed at autopsy	1 (0.6)

**Table 3:** Characteristics of the 70 patients with locally advanced or metastatic bladder cancer surviving for more than 3 months post diagnosis.

Characteristics	Unit
Median age at diagnosis, years (IQR)	78 (73–83)
Gender, *n* (%)	
Male	46 (65.7)
Female	24 (34.3)
ASA physical status at diagnosis, *n* (%)	
1	3 (4.3)
2	30 (42.9)
3	36 (51.4)
4	1 (1.4)
Median BMI at diagnosis, kg/m^2^ (IQR)	24.9 (23.1–28.12)
Median creatinine level at diagnosis, μmol/L (IQR)	111 (83;136)
Comorbidity, *n* (%)	
Diabetes mellitus without complications	15 (28.8)
Chronic lung disease	15 (28.8)
Myocardial infarction	12 (23.1)
Pulmonary embolism	8 (15.4)
Renal disease (moderate/severe)	8 (15.4)
Peripheral vascular disease	8 (15.4)
Cerebrovascular disease	8 (15.4)
Other cancer disease, non-metastatic	7 (13.5)
Other cancer disease, metastatic	4 (7.7)
Dementia	3 (5.8)
Liver disease	2 (3.8)
Diabetes mellitus with complications	2 (3.8)
Connective tissue disease	1 (1.9)
Gastric ulcer	1 (1.9)
Hemiplegia	1 (1.9)
Chronic heart disease (EF < 40%)	1 (1.9)
Lymphoma	1 (1.9)
TNM stage of metastatic disease, *n* (%)	
Any T, N+, M0	14 (20)
Any T, Any N, M1	55 (78.6)
Metastasis sites, *n* (%)	
Lymph nodes	38 (54.3)
Lungs	20 (28.6)
Bone	18 (25.7)
Liver	14 (20.0)
Soft tissue	8 (11.4)
Other	8 (11.4)
CNS	4 (5.7)
Local recurrence	3 (4.3)
Carcinomatosis	2 (2.9)
Initial presentation of urothelial disease, *n* (%)	
Metastatic or locally advanced bladder cancer	20 (28.6)
Local bladder cancer	50 (71.4)

**Table 4:** Data for the 50 patients initially diagnosed with local disease.

Data	Unit
Median time from primary local urothelial disease to metastatic disease, days (IQR)	340 (201; 828)
Prior urothelial disease, TNM stage, *n* (%)	
Ta	4 (8.0)
T1	3 (6.0)
T2	17 (34.0)
T3	21 (42.0)
T4a	5 (10.0)
N0	36 (72.0)
N1	13 (26.0)
Nx	1 (2.0)
M0	47 (94.0)
M1	3 (6.0)
Prior urothelial disease, treatment, *n* (%)	
Radical Cystectomy	31 (62.0)
Non-radical cystectomy	5 (10.0)
Curative radiotherapy	8 (16.0)
TURBT, intravesical therapy	5 (10.0)
No treatment	1 (2.0)
Prior urothelial disease, neoadjuvant chemotherapy, *n* (%)	7 (14.0)
Metastatic pattern for prior treated patients, *n* (%)	
Lymph nodes	37 (74.0)
Lungs	20 (40.0)
Bones	18 (36.0)
Liver	14 (28.0)
Soft tissue	8 (16.0)
Other	6 (12.0)
CNS	4 (8.0)
Local recurrence	3 (6.0)
Carcinomatosis	2 (4.0)

## Data Availability

Due to privacy and ethical considerations, the supporting data is not available, however, limited anonymous data can be shared upon request.
